# Acknowledgement to Reviewers of *Pathogens* in 2013

**DOI:** 10.3390/pathogens3010185

**Published:** 2014-02-26

**Authors:** Pathogens Editorial Office

**Affiliations:** MDPI AG, Klybeckstrasse 64, CH-4057 Basel, Switzerland

The editors of *Pathogens* would like to express their sincere gratitude to the following reviewers for assessing manuscripts in 2013:

Allen, David J.
Andremont, Antoine
Antonov, I.
Atkinson, Robyn
Ayala-Del-Río, Héctor L.
Babu, Subash
Baltrus, David
Barbet, Anthony
Baron, Thierry G.
Bessen, Richard
Biagi, Elena
Bjarnsholt, Thomas
Boone, David
Bowman, John P.
Boylan, J.
Bradford, B. J.
Brayton, Kelly A.
Broder, Christopher
Buccigrossi, V.
Budischak, Sarah
Capozzoa, Alejandra Victoria
Castilla, Joaquin
Catry, Boudewijn
Cavalieri, Francesca
Charroux, Bernard
Cho, Sang-Nae
Chu, Justin J. H.
Cialla, Dana
Cohen, Marc
Cordeiro, Yraima
D’auria, Giuseppe
Dey, Bappaditya
Do, Thuy
Donelli, Gianfranco
Drebot, Michael
Fava, Francesca
Fernando, Samodha C.
Fischer, Wolfgang
Fournier, Pierre-edouard
Gambetti, Pierluigi
Ganta, Roman
Gause, William C.
Gillevet, Patrick
Glatzel, M.
Gobet, Angélique
Goldsmith, Jason
Gomes, Joana
Gomez-Llorente, Carolina
Grandadam, Marc
Gray, Wayne
Gubler, Duane
Guttman, Julian A.
Haïk, Stéphane
Hao, Pei
Head, Mark
Helmby, Helena
Hershfield, Jeremy
Holbrook, Michael
Hope, Jim
Hurdle, Julian
Ike, K.
Jesús, Requena
Jiménez-Ruiz, Elena
Johnson, Reed F.
Kemmerling, U.
Kinchington, Paul
Knight, Rob
Kosik-Bogacka, Danuta I.
Kovacs, Gabor
Labruyere, Elisabeth
Larsen, Lone Heimann
Lass-Flörl, C.
Lo, Michael
Lulich, Jody
Maeda, Shin
Maresso, Anthony
Martinez-Sobrido, Luis
Martinez, Jose Luis
Mathey-Prevot, Bernard
Matthias, Michael A.
McDonald, L. Clifford
McLean, Catriona
Mcmurray, David N.
Mehaffy, Carolina
Midtvedt, Tore
Minton, J. Ernest
Mizoguchi, Atsushi
Mollapour, Mehdi
Montagne, Lucile
Moore, Ben
Moore, D. P.
Moreno, J. J.
Murray, Paul G.
Musch, Mark
Nava, Porfirio
Nishida, Noriyuki
Niyonsaba, François
Oliver, W. T.
Lauber, Christian L.
Lee, Hyoung-gon
Lin, Jin-Ming
Othman, Ahmad
Pagano, Joseph S.
Peek, Richard M.
Petri, Daniel
Pillar, Dylan
Prabha, Vijay
Reichel, Michael
Reimer, Aleisha
Rejmanek, Daniel
Reljic, Rajko
Rey, Federico
Rockx, Barry
Röhrl, Johann
Romalde, Jesús López
Rubenstein, Richard
Růžek, Daniel
Sarris, Panagiotis F.
Schares, Gereon
Schaub, Günter A.
Schaudies, R. Paul
Schmid-Hempel, Paul
Scorpio, Diana G.
Scortichini, Marco
Serra, A.
Serrano-Luna, Jesús
Seuberlich, Torsten
Sly, Laura
Snyder, Chris
Sonawane, Avinash
Sørensen, Søren Johannes
Stack, Mick
Sukumaran, Bindu
Tang, Chuan Y.
Tsou, Lun
Van Beek, J.
Vilette, Didier
Walker, David
Walter, Jens
Wang, Dan
Wang, Fei
Warawa, Jonathan
Whitehouse, Michael
Williams, Dustin L.
Wong, K. T.
Yang, Liang
Yeh, Yi-Chun
Yin, Hang
Yokoyama, Takashi
Yu, Xuejie
Zerr, Inga
Zilbauer, Matthias

